# Second passage experiments of chronic wasting disease in transgenic mice overexpressing human prion protein

**DOI:** 10.1186/s13567-022-01130-0

**Published:** 2022-12-16

**Authors:** Brent Race, Chase Baune, Katie Williams, James F. Striebel, Andrew G. Hughson, Bruce Chesebro

**Affiliations:** Laboratory of Persistent Viral Diseases, Rocky Mountain Laboratories, National Institute of Allergy and Infectious Diseases, National Institutes of Health, 903 South Fourth Street, Hamilton, MT 59840 USA

**Keywords:** RT-QuIC, prion, cross-species transmission, barrier, chronic wasting disease, transgenic mice

## Abstract

Chronic wasting disease (CWD) is a prion disease of cervids including deer, elk, reindeer, and moose. Human consumption of cervids is common, therefore assessing the risk potential of CWD transmission to humans is critical. In a previous study, we tested CWD transmission via intracerebral inoculation into transgenic mice (tg66 and tgRM) that over-expressed human prion protein. Mice screened by traditional prion detection assays were negative. However, in a group of 88 mice screened by the ultrasensitive RT-QuIC assay, we identified 4 tg66 mice that produced inconsistent positive RT-QuIC reactions. These data could be false positive reactions, residual input inoculum or indicative of subclinical infections suggestive of cross species transmission of CWD to humans. Additional experiments were required to understand the nature of the prion seeding activity in this model. In this manuscript, second passage experiments using brains from mice with weak prion seeding activity showed they were not infectious to additional recipient tg66 mice. Clearance experiments showed that input CWD prion seeding activity was eliminated by 180 days in tg66 mice and PrPKO mice, which are unable to replicate prion protein, indicating that the weak positive levels of seeding activity detected at later time points was not likely residual inoculum. The failure of CWD prions to cause disease in tg66 after two sequential passages suggested that a strong species barrier prevented CWD infection of mice expressing human prion protein.

**Supplementary Information:**

The online version contains supplementary material available at 10.1186/s13567-022-01130-0.

## Introduction

Chronic wasting disease (CWD) is a prion disease, or transmissible spongiform encephalopathy (TSE), that causes a fatal neurogenerative disease in members of the cervid family including deer, elk, moose and reindeer. CWD was initially described in the late 1960’s in captive deer from Colorado and Wyoming and is now widespread in both wild and farmed cervids of North America [1, 2]. South Korea, Norway, and Finland have also reported CWD, but the reported prevalence in these locations is much lower [3]. At this point, eradication of CWD from endemic areas is extremely unlikely, as mis-folded prions such as CWD are very stable in the environment [4, 5], and the infectious dose required for transmission to additional deer is extremely low [6]. As CWD continues to spread across North America and other countries, the number of humans consuming CWD-infected meat and cervid products will increase. Fortunately, to date, there have been no confirmed reports of CWD transmission to humans [7]. However, understanding the relative risk that CWD poses to human health remains important to protect and educate consumers of cervids and cervid-derived products.

Research into whether CWD can infect humans has primarily focused on three topics: epidemiologic studies, in vitro conversion models and in vivo transmission models [7]. A few epidemiologic studies have been completed, and several are ongoing. The focus of these studies is to look for evidence of CWD transmission to humans. Such evidence may come in the form of finding an increase in prion disease among venison consumers living in CWD endemic areas, or any anomaly in prion disease age at onset in at risk populations. Fortunately, an increase in overall prion disease rates among venison consumers, or reports of an unexplained increased incidence of young people developing prion disease have not been reported to date.

In vitro and in vivo models have also been developed to test the strength of the cervid CWD to human transmission barrier. In vitro studies have demonstrated that a strong barrier exists, but it has not been absolute in all studies [7]. In vivo studies have used non-human primate models and transgenic mice to assess the CWD species barrier. Squirrel monkeys were susceptible to CWD but cynomolgus macaques were not [8–11]. Macaques are a closer genetic match to humans overall and have traditionally been susceptible to most human-tropic prion diseases, so the observation that macaques are resistant to CWD infection is encouraging. Transgenic mice genetically engineered to express human prion protein have proved to be an excellent tool for studying prion disease transmission. To date, eight groups including ours have tested the susceptibility of human prion protein-expressing mice to CWD infection [12–19]. Seven groups have reported no strong evidence for CWD transmission to humanized mice [12–18]. A recent publication by Hannaoui et al. reported bioassay evidence for transmission, but initial classification of prion infection was based heavily on clinical signs rather than consistent biochemical confirmation of prion infection [19].

Our recent study found that 95% of the human prion protein expressing mice we tested had no evidence for CWD transmission, but 4 out of 88 mice screened for prion seeding activity using the ultrasensitive RT-QuIC assay did have seeding activity above baseline values [18]. In the initial publication, we classified these mice as indeterminate since they were not negative, nor were they convincingly positive [18]. We were uncertain whether the weak prion seeding activity detected was due to a subclinical prion infection, residual cervid CWD inoculum, false positive reactions or spontaneous transformation/aggregation of normal prion protein. In the current paper, we did additional experiments to better understand the source of the weak seeding activity. Second passage experiments into tg66 and tg33 mice were completed to determine if the weak seeding activity represented bona fide infectivity, and to provide additional time for CWD to adapt a human tropism. CWD clearance experiments were also performed to better understand how long input CWD can persist in brain following inoculation. Our results showed that the seeding activity observed in our initial experiments was likely not input inoculum and that brain material from weakly positive mice was not infectious on second passage to transgenic mice expressing human prion protein, suggesting the existence of a strong species barrier between cervid CWD and humans.

## Materials and methods

### Experimental mice

All mice were housed at the Rocky Mountain Laboratory (RML) in an AAALAC accredited facility in compliance with guidelines provided by the Guide for the Care and Use of Laboratory Animals (Institute for Laboratory Animal Research Council). Experimentation followed RML Animal Care and Use Committee approved protocol #2018–052. Generation of tg66 transgenic mice expressing human PrP were described previously [9]. Tg66 mice were originally made by Richard Rubenstein and provided to RML by Robert Rohwer. Tg66 mice are on an FVB/N genetic background and are homozygous for a transgene that encodes human prion protein M129. Tg66 mice overexpress human PrP at 8–16-fold levels higher than normal physiologic levels and have been shown to be susceptible to vCJD, sCJD and mouse-adapted 22L scrapie [9, 20]. Tg66 mice do not express any mouse PrPC. Tg33 mice express mule deer prion protein at levels at 1–2 × physiologic levels and their construction has been described previously [21]. Tg33 mice also do not express any mouse PrPC. The PrPKO mice used in this study have been described previously as C57BL10/SnJ-Prnp-/- [22, 23] and do not express any prion protein. Since those publications, additional breeding, backcrossing and single nucleotide polymorphism (SNP) genotyping has been performed to decrease the amount of 129/Ola flanking genes surrounding the neomycin cassette disrupted Prnp gene from 47.4 mB to approximately 19.1 mB.

### Inocula, inoculations and experimental design for the second passage experiments

To determine whether CWD had caused a subclinical infection in a subset of tg66 mice we performed second passage experiments in two strains of recipient transgenic mice (tg66 and tg33). Nine tg66 “donor” mouse brains were inoculated into groups of 7–12 recipient mice of each strain (Table 1). Four donor mouse brains selected for second passage were previously classified as indeterminate based on RT-QuIC data, and five donor mice were previously classified as negative. Donor brain homogenates were diluted to a 1% w/v concentration using phosphate buffered balanced saline supplemented with 2% fetal bovine serum and a 30 µL volume was inoculated into the left-brain hemisphere of anesthetized recipient mice. Following inoculation, mice were observed for onset of clinical signs as described below. At the time of euthanasia, brains were collected for RT-QuIC analysis.

**Table 1:** **Second passage of CWD-inoculated tg66 mouse brain**

Tg66 donor brain^a^	Recipient mouse strain	DPI range at euth	TSE suspect^b^	RT-QuIC^c^
*B378-3* *717 dpi* *19/32 RT-QuIC*	*Tg66*	*627–700*	*5/12*	*0/12*
	*Tg33*	*552–700*	*2/12*	*0/12*
*B349-1* *651 dpi* *7/24 RT-QuIC score*	*Tg66*	*329–700*	*3/12*	*0/12*
	*Tg33*	*501–700*	*3/12*	*0/12*
*B351-3* *662 dpi* *4/12 RT-QuIC score*	*Tg66*	*532–700*	*1/12*	*0/12*
	*Tg33*	*587–700*	*2/10*	*0/10*
*B377-4* *710 dpi* *14/24 RT-QuIC score*	*Tg66*	*452–700*	*1/12*	*0/12*
	*Tg33*	*378–700*	*0/11*	*0/11*
B378-4 717 dpi 0/4 RT-QuIC score	Tg66	469–673	1/8	0/8
	Tg33	572–700	0/7	0/7
B348-3 635 dpi 0/4 RT-QuIC score	Tg66	657–700	2/8	0/8
	Tg33	679–700	0/7	0/7
B354-4 635 dpi 0/4 RT-QuIC score	Tg66	612–700	7/10	0/10
	Tg33	553–700	6/10	0/10
B380-1 626 dpi 0/4 RT-QuIC score	Tg66	480–700	1/9	0/9
	Tg33	519–700	0/8	0/8
B351-1 662 dpi 0/4 RT-QuIC score	Tg66	463–653	1/10	0/10
	Tg33	404–700	1/8	0/8
		Days old		
Uninoculated	Tg66	257–735	1/9	0/9
	Tg33	288–572	0/5	0/5

^a^ Donor brain tissues were obtained from mice previously classified as indeterminate (weak positive) or negative. The dpi and original RT-QuIC scores are provided for reference. Indeterminate mice are in italics.

^b^ Mice that showed a decrease in body condition (weight loss), weakness and/or neurologic signs (ataxia, gait abnormalities or balance issues) were classified as TSE suspects based on overall assessment of the live mouse.

^c^ The RT-QuIC assay was used to screen all second passage mice for prion seeding activity. The number of positive mice over the number of mice tested is shown. Mice were scored positive if ≥ 50% of the assay wells were positive. Positive controls were run on each assay plate and were consistently 100% positive (not shown in table).

### Inocula, inoculations and experimental design for the clearance experiments

To better understand the clearance kinetics of CWD following inoculation, we intracerebrally inoculated three different strains of genetically modified mice with the two different CWD brain homogenate inocula that created indeterminate mice in our original study (WTD-1 and Elk-2) [18]. Each mouse inoculated with WTD-1 or Elk-2 received 1.2 × 10^5^ or 6.0 × 10^4^ LD_50_ respectively per injection. The three mouse strains tested were: 1. Tg66 mice that over-express express human prion protein 2. Tg33 mice that express deer prion protein at levels slightly higher than mule deer and are highly susceptible to CWD infection and 3. PrPKO mice that have no prion protein expression and are unable to amplify prions. Tg33 mice provided a positive control, PrPKO mice provided a no replication control and baseline clearance rate in the absence of PrP expression. Tg66 mice represent our experimental mouse model used in our original transmission paper [18].

Each mouse was anesthetized with isoflurane and inoculated intracerebrally in the left hemisphere with 30 µL of a 1% CWD brain homogenate using a 27-gauge needle. Following inoculation of CWD, groups of inoculated mice were euthanized at several different time-points including 7, 28, 90, 180, 360 and 675 dpi (N = 4–7 per strain per timepoint) and the left hemisphere of each brain was removed and screened by RT-QuIC to measure prion seeding activity. Tg33 mice were only tested at timepoints out to 180 days, as they develop terminal disease around 300 days and would be expected to continue to give strong positive RT-QuIC results beyond 180 days. Normal uninfected control tissues were collected from 5 tg33 mice that were 288–572 days old, 9 tg66 mice that were 257–735 days old and 5 PrPKO mice that were 127 days old.

### Clinical observations

All mice in both the clearance study and second passage experiments were observed once daily by animal care staff and 3–5 times per week by prion investigators for assessment of overall health and observation for neurologic signs consistent with prion infection. In both studies, mice not designated for early time-points, were observed for nearly their entire natural lifespan. Mice were euthanized when they developed conditions necessitating a humane end-point (e.g. cancer, dermatitis, respiratory difficulty, chronic ocular lesions) or for weight loss and/or neurologic signs consistent with neurologic disease. Very few mice developed signs of neurologic disease or wasting, and the mice that did were scattered throughout several groups (Additional file 1). Mice that did not develop clinical and/or age-related issues requiring euthanasia were electively euthanized at day 675 in the clearance study and from 665 to 700 days post inoculation in the second passage experiments. Following euthanasia, brains were removed, cut in half along the midline and frozen for later use in diagnostic assays. Details on the dpi, clinical status and RT-QuIC results for each individual mouse from the second passage experiments can be found in Additional file 1.

### RT-QuIC

RT-QuIC reactions were performed as previously described using recombinant hamster 90–231 (Ha rPrP) (accession no. KO2234) as the substrate [18, 24]. Briefly, brains were homogenized, diluted to 10% and cleared with a one-minute centrifugation at 2000 rcf. Supernatants were then serially diluted in 0.1% SDS (sodium dodecyl sulfate, Sigma)/PBS/N2 (Gibco) to yield 10^–3^ brain tissue concentrations. Brain samples were tested only at the 10^–3^ dilution as higher concentrations of brain homogenate can lead to inhibition of the assay [24, 25]. 2 µL sample volumes were added to reaction wells of a black 96-well, clear bottom plate (Nunc) containing 98 µl of RT-QuIC reaction mix, resulting in final concentrations of 0.002% SDS, 10 mM phosphate buffer (pH 7.4), 300 mM NaCl, 0.1 mg/mL rPrPsen substrate, 10 μM thioflavin T (ThT), 1 mM ethylenediaminetetraacetic acid tetrasodium salt (EDTA). The plate was then sealed with a plate sealer film (Nunc) and incubated at 50 °C in a BMG FLUOstar Omega plate reader with a repeating protocol of 1 min shaking (700 rpm double orbital) and 1 min rest throughout the indicated incubation time. ThT fluorescence measurements (450 ± 10 nm excitation and 480 ± 10 nm emission; plate bottom read) were taken every 45 min.

The plate reader gain was consistent for each run, as were the concentrations of thioflavin T and SDS in each reaction. The maximum fluorescence readout on our plate reader is 260 000 units. For all runs, the gain was set at 1600. Four replicate wells from the same positive control mouse were run on each plate (tg33 #NM462 at a 10^–3^ dilution). In addition, 4–12 negative control wells were run at a 10^–3^ on each plate. To be consistent with our initial paper [18] we used a 25-h cut-off time for the assay. Individual wells were considered positive if they reached a fluorescence level greater than 10% of the average fluorescent values measured for the positive control wells at the 25-h time-point. Compared to baseline negative control wells, an increase in fluorescence from baseline to reach levels equivalent to the 10% value of positive control samples was typically 20–40 standard deviations above baseline fluorescence levels. Individual mice were scored positive if ≥ 50% of the individual assay wells were positive.

**Figure 1:**
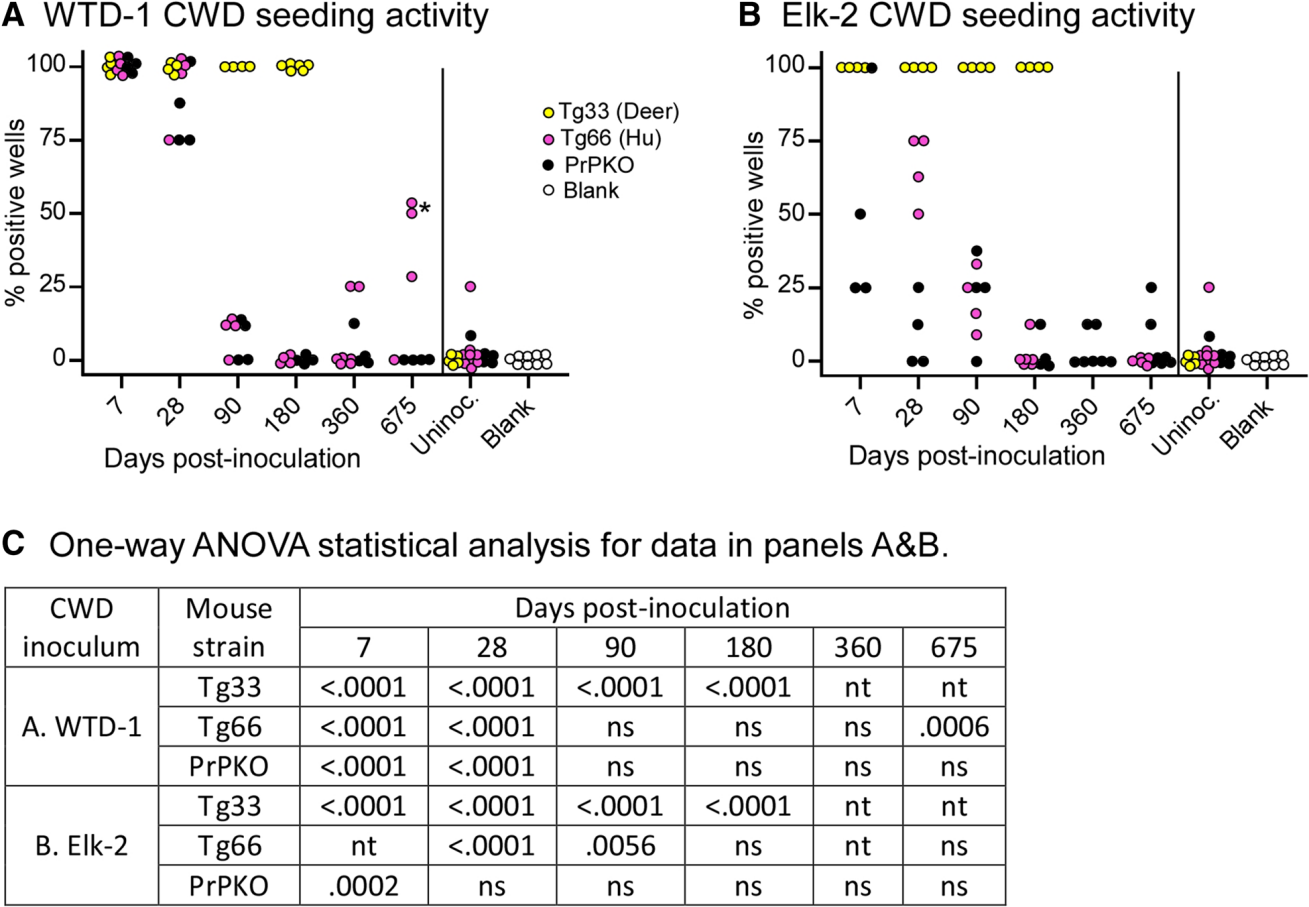
**Clearance of prion seeding activity at various times following intracerebral inoculation of CWD.** Panels A&B, RT-QuIC data showing the percentage of assay wells positive for prion seeding activity at several times post intracerebral inoculation with WTD-1 (**A**) or Elk-2 (**B**) CWD. Mouse strains are indicated with different colored circles, each circle represents data from an individual mouse brain. Each brain was assayed using a minimum of 4 replicate wells for the 7 and 28 day time-points and an 8 well minimum at all time points of 90 days and longer. The asterisk in panel A indicates two mice that were statistically different (*P* < 0.05) versus uninoculated controls using the Fisher’s exact T-test. C. Additional statistical analysis was performed using Dunnett’s multiple comparisons test (One-way ANOVA). Strains of mice were tested independently, all time-points within a strain were compared to the uninoculated control mice of that specific strain. *P*-values that were < 0.05 are provided; ns, not significant; nt, not tested. The uninoculated mice shown are from each of the three mouse strains tested and mice were euthanized at several different times. Age information for these control mice can be found in Additional file 2. In the blank column, each circle represents data from quadruplicate RT-QuIC assay reactions that were not seeded with any brain homogenates and included as controls for spontaneous reactions from the substrate.

For the clearance experiments, each brain collected at the 7, 28 and 90 day time points was screened with four independent wells tested at a 10^–3^ brain homogenate dilution. Brains collected at 180, 360, and 675 dpi were screened on a minimum of two separate plates, on different days, for a total of 8–16 wells per brain. The percentage of positive wells per sample are shown in Figure 1. Brains from the second passage experiment tg33 and tg66 mice were screened using 4–12 wells per mouse (Additional file 1).

## Results

### Second passage of indeterminate CWD-inoculated tg66 mice

Sequential passage of prion agents into additional recipients is a useful method to determine the strength of species barriers. This procedure provides additional time for the prion to adapt and amplify to higher levels in a new host. To determine whether brain tissue from CWD-inoculated mice from our first passage experiments harbored subclinical prion infection, we performed second passage inoculations into tg66 and tg33 mice. Tg66 mice over-express human prion protein and were inoculated to determine whether the low levels of RT-QuIC positivity observed on first passage represented bona fide human tropic prion infectivity.Tg33 mice, which express deer prion protein were inoculated to test for residual inoculum, non-adaptive prion amplification [26], or the potential of a dual tropic prion agent.

Brain homogenates from four RT-QuIC indeterminate and five RT-QuIC negative tg66 first passage mice were inoculated into groups of both tg66 and tg33 recipient mice (Table 1). Following inoculation, tg66 and tg33 mice were monitored for onset of neurologic signs and/or wasting. As mice aged, several mice did show a decrease in body condition, occasionally in combination with weakness and mild ataxia. Based on CWD observed in deer and cervidized mice, these signs could be consistent with prion disease, and these mice were classified as TSE suspects (Table 1). This low incidence of suspect mice was not strikingly different than those observed in previous tg66 mouse studies [18, 27] and not entirely unexpected in mice reaching nearly two years of age. It was rare to see more than just a few mice from each group develop signs consistent with prion disease (Table 1 and Additional file 1). Importantly, weight loss, weakness and gait abnormalities are rather non-specific signs, and by themselves should not be considered diagnostic of prion disease.

To screen mice for confirmation of prion disease, we used the RT-QuIC assay. Brain homogenates from every tg66 and tg33 second passage mouse were screened on a minimum of four replicate wells. Of the 178 s passage recipient mice screened, no mouse had positive prion seeding activity in > 50% of the wells screened (Table 1 and detailed results in Additional file 1). None of the uninoculated tg66 mice scored positive by the  >50% well criteria, but a few mice had positive wells (1.3% of the reaction wells tested were positive). Collectively, the second passage recipient tg66 mice were positive in 1.5% of the reaction wells (Calculated from Additional file 1). If mice were showing clinical signs because of prion infection, we anticipated a strong positive result by RT-QuIC analysis. Indeed, brain tissue from rodents, cervids or humans with clinical prion disease can typically be diluted by at least 100 000-fold before RT-QuIC positivity is lost [18, 24]. Our results were to the contrary, and the passage data from all four indeterminate mice and five negative mice suggested no prion infectivity was present. This evidence showed that CWD was not readily adapting into a human-tropic prion agent, nor was any residual deer-tropic CWD infectivity detected.

### CWD-prion clearance following intracerebral inoculation

To better understand the clearance and replication of CWD following intracerebral injection, we inoculated tg66, tg33 and PrPKO mice with the two different CWD inocula (WTD-1 and Elk-2) that generated weak positivity in our previous study. Understanding the duration of input prion infectivity or seeding activity in brain following inoculation is critical toward distinguishing newly formed prions versus residual input prions. Following inoculation, mice were euthanized at several time points and screened by RT-QuIC for prion seeding activity (Figure 1 and Additional file 2). Following infection with either CWD source, all tg33 mice had strong seeding activity in 100% of the wells tested by RT-QuIC at 7, 28, 90 and 180 dpi (Figure 1A, B yellow circles). This was expected, as tg33 mice express deer prion protein and are susceptible to CWD infection. In contrast, PrPKO mice, which are unable to amplify prions, showed a steep decline in detectable prion seeding activity from 7 to 180 dpi. At 180, 360 and 675 dpi, a few PrPKO mice continued to show seeding activity in a low percentage of wells, but these data were not statistically different than seeding activity measured in uninfected PrPKO mice (Figure 1C).

Initial clearance of CWD seeding activity in tg66 mice was similar to the clearance rate observed in PrPKO mice, with the lowest level of seeding activity detectable in tg66 mice at 180 dpi (Figures 1A and B pink circles). These low levels of seeding measured at both 180 and 360 dpi were statistically equivalent to uninfected tg66 mice (Figure 1C). Five tg66 mice inoculated with Elk-2 remained low throughout the latest time-point at 675 dpi (Figure 1B). In contrast, tg66 mice inoculated with WTD-1, and tested at 675 dpi showed higher levels of prion seeding activity compared to uninfected tg66 mice (Figures 1A and C). Further analysis of these mice was performed on an individual basis by comparing individual well data from CWD-inoculated mice to uninfected tg66 mice using a Fisher’s exact T-test. Two of the four mice from 675 dpi were statistically different than uninfected controls by this analysis (Figure 1A asterisks). These mice with weakly positive results were like the four mice described as indeterminate in our initial study [18], where classification as a positive or negative was difficult.

## Discussion

Our initial studies were designed to test potential CWD cross-species transmission to humans by using transgenic mice (tgRM and tg66) that express human prion protein as experimental models. The preliminary conclusion from the original work was that CWD did not infect the transgenic mice, but the results were not entirely clear [18]. Four tg66 mice that showed no evidence of prion infection based on immunohistochemistry or immunoblot were partially positive by the RT-QuIC, an ultrasensitive prion seeding detection assay. Although this partial positivity was close to the assay detection limit and several orders of magnitude lower in seeding activity than is found in the brains of individuals with clinical CWD or CJD, these mice were classified as indeterminate, as we were uncertain whether the prion seeding activity observed was due to subclinical prion infection, residual input from the CWD positive inoculum, spontaneous (CWD-independent) formation of prion seeding activity in aging PrP-overexpressing mice, or false positive reactions. To follow-up on these hypotheses, we performed second passage and CWD clearance experiments.

Historically, serial passage of prions has been used to determine prion infectivity, test the strength of species barriers (transmissibility) and characterize adapted prions in a new host [28–30]. In our study, we performed second passage of brain homogenates derived from 9 CWD-inoculated first passage tg66 donor mice, four of which had been previously classified as RT-QuIC indeterminate. Our second passage experiments used two strains of recipient mice, tg66 (human PrP) and tg33 (deer PrP) so that we could define the species tropism of the adapting (or non-adapting) prions [26, 31, 32]. We found no evidence for transmission of prion disease to any of our second passage tg66 or tg33 recipient mice (Table 1). The failure of CWD to adapt to a human tropic prion agent after two serial passages in tg66 mice indicates a strong species barrier inhibits CWD prion conversion of human prion proteins. The indeterminate levels of seeding activity from our initial experiments were either below the level required for transmission or did not represent bona fide infectious prions. Previous studies have shown that the RT-QuIC is more sensitive than bioassay, and able to detect sub infectious levels of prions [24, 33–35].

Our negative transmission data is consistent with many other groups that have performed single passages of natural sources of CWD into transgenic mice that express human prion protein [12–18]. In contrast, our data differs from two other groups. Wang et al. used CWD seeded PMCA products as inocula and reported transmission to 100% of the inoculated transgenic mice that expressed human prion protein [36]. It is unclear how representative the PMCA replication process is compared to natural conditions. A second very recent study has reported clinical disease in several CWD-inoculated tg650 mice [19]. Of concern, these clinical mice did not have consistent positive confirmatory tests for prion disease including immunohistochemistry, immunoblot, and RT-QuIC. Typically, mice that develop end stage clinical prion disease would have robust evidence for prion accumulation.

Results from our clearance experiments were informative and showed that input CWD prion seeding activity decreased quickly in both tg66 and PrPKO mice and reached negligible levels by 180 dpi (Figures 1A and B). This data suggested that residual inoculum was not an explanation for the seeding activity measured in indeterminate mice unless the presence of PrP^C^ somehow retards clearance of the inoculum even without its conversion to new prion seeding activity. In PrPKO mice the measured seeding activity remained low for the duration of the study from 180 to 675 dpi with a calculated average well positivity rate of 2.7% (7/256 positive wells, summed data from Additional file 2). This low level of positive wells was statistically equivalent to the 1.8% rate measured in PrPKO uninoculated mice. The decrease in prion seeding activity in PrPKO mice was not surprising, as PrPKO mice lack prion protein, and are unable to become infected with prion diseases leading to formation of additional prion seeding activity. In tg66 mice, the data was less clear because of additional indeterminate mice being identified at 675 dpi.

The rate of prion clearance we observed in PrPKO mice was similar to reports from Bueler et al., Prusiner et al. and Sailer et al. that showed a rapid decrease to below bioassay detection limits within days-weeks following inoculation of rodent scrapie prions [37–39]. Longer clearance times were shown by Race et al. where they reported hamster scrapie infectivity persisted in PrPKO mice up to 217 days, but not at 330 days [40]. Clearance of CWD and RML scrapie prions were reported after 500 days post-inoculation in a non-permissive host, but detailed timepoints prior to 500 days were not tested [26]. Beringe et al. reported the longest prion persistence in PrPKO mice using sCJD. Their data showed that both infectivity and RT-QuIC seeding activity remained after 450–700 days post-inoculation [41]. Clearly, persistence of prions following inoculation can vary greatly depending on the strain and titer of prions in the inoculum, and these factors must be considered when evaluating transmission results.

The likelihood of false positive RT-QuIC assay results explaining the seeding activity in indeterminate mice seems unlikely based on data from our uninfected control mice and the cohort of 93 s passage tg66 mice. Individual well data from uninoculated PrPKO and tg66 mice had 1/56 and 1/80 positive wells for calculated false positive well rates of 1.8% and 1.3% respectively. Based on 136 test wells from uninfected control mice, the false positive rate for individual well reactions in our assay was approximately 1.5%. Mathematically, using a 1.5% occurrence rate and four replicate reactions, we determined the cumulative binomial probability for a normal sample to have ≥ 50% positive wells was 0.132%. This extremely low probability suggests that the near 50% positive well rate observed in the indeterminate mice were unlikely a result of false positive reactions. In addition to our uninoculated control mice, the calculated positive well percentage from all 93 of the tg66 mice inoculated in the second passage experiments was also 1.5% (6 positive wells / 392 wells tested) (Additional file 1). This entire group of 93 s passage recipient tg66 mice did not develop prion disease and the majority were electively euthanized at > 600 dpi. No mouse had positive RT-QuIC well data sufficient to classify them as positive or indeterminate, with only a very rare positive well reaction (1.5% of the wells) (Additional file 1). This additional observation of extremely rare positive wells in 93 aged tg66 mice provides support that the seeding activity measured in first passage indeterminate mice was not due to false positive reactions.

In conclusion, our current study confirmed that even on second passage, CWD-inoculated, humanized mice did not develop prion disease. Our findings have also allowed us to eliminate most of our previous explanations for indeterminate RT-QuIC data. Neither residual inoculum nor false positive reactions in the RT-QuIC are probable explanations. Subclinical infections following a single passage remained a possibility. However, no evidence for transmission was found following second passage. It is not clear whether this failure to transmit was due to an insufficient level of infectivity, or a result of poorly adapted or spontaneously transformed prions that were unable to propagate infection within the lifespan of a mammalian host. Regardless of the explanation, tg66 mice in our experiments appear to be a dead-end host for CWD transmission. Despite this encouraging result, caution is still warranted regarding human consumption of CWD-infected cervids. Our studies in the laboratory are limited, and it is nearly impossible to test all the potentially different combinations of CWD strains against the many variations of human prion genotypes.

## Supplementary Information

**Additional file 1. Detailed information for individual second passage recipient mice.** Additional file 1 includes a table with observation periods, clinical signs, and RT-QuIC assay individual well data for all second passage mice.

**Additional file 2. Detailed information for individual clearance study mice.** Additional file 2 includes a table that provides RT-QuIC assay individual well data for transgenic mice included in the CWD clearance study. Data from three strains of transgenic mice inoculated with CWD are shown at time-points from 7 to 675 days post-inoculation [30].

## Data Availability

The dataset supporting the conclusions of this article are included within the article and the two additional files. The brain tissue homogenates from these experiments are available from the corresponding author on request.
