# Supplementary material for: Second passage experiments of chronic wasting disease in transgenic mice overexpressing human prion protein
Source: Vet Res. 2022 Dec 16;53:111. doi: 10.1186/s13567-022-01130-0 (PMC9758843; doi:10.1186/s13567-022-01130-0)
Supplement: Supplementary file 1 — Additional file 1. Detailed information for individual second passage recipient mice. Additional file 1 includes a table with observation periods, clinical signs, and RT-QuIC assay individual well data for all second passage mice. [file 13567_2022_1130_MOESM1_ESM.docx]

**Additional file 1. Detailed information for individual second passage recipient mice.**

| **Donor Brain** | **Recipient Mouse Strain** | **DPI @ euth.** | **Clinical Signs^1^** | **RT-QuIC^2^** |
| --- | --- | --- | --- | --- |
| B378-3  717 DPI  19/32 RTQ score | Tg66 | 700 | Y | 0/4, 0/4 |
|  |  | 700 | Y | 0/4, 0/4 |
|  |  | 700 | Y | 0/4, 0/4 |
|  |  | 700 | Y | 0/4 |
|  |  | 700 | N | 0/4 |
|  |  | 700 | N | 0/4 |
|  |  | 627 | N | 0/4 |
|  |  | 648 | N | 0/4 |
|  |  | 700 | N | 1/4 |
|  |  | 657 | Y | 0/4 |
|  |  | 657 | N | 0/4 |
|  |  | 657 | N | 1/4 |
|  | Tg33 | 552 | Y | 1/4, 0/4 |
|  |  | 653 | N | 0/4 |
|  |  | 653 | N | 0/4 |
|  |  | 665 | N | 0/4 |
|  |  | 665 | N | 0/4 |
|  |  | 665 | N | 0/4 |
|  |  | 700 | N | 1/4, 0/4 |
|  |  | 700 | N | 0/4, 0/4 |
|  |  | 700 | N | 1/4, 1/4 |
|  |  | 577 | Y | 0/4 |
|  |  | 651 | N | 0/4 |
| B349-1  651 DPI  7/24 RTQ score | Tg66 | 329 | N | 0/4 |
|  |  | 561 | Y | 0/4 |
|  |  | 700 | N | 0/4 |
|  |  | 645 | N | 0/4 |
|  |  | 645 | N | 0/4 |
|  |  | 645 | N | 0/4 |
|  |  | 494 | N | 0/4 |
|  |  | 670 | N | 1/4, 0/4 |
|  |  | 670 | N | 0/4 |
|  |  | 700 | Y | 0/4 |
|  |  | 700 | Y | 0/4 |
|  |  | 700 | N | 0/4 |
|  | Tg33 | 700 | N | 0/4 |
|  |  | 700 | N | 0/4 |
|  |  | 665 | N | 0/4 |
|  |  | 501 | Y | 0/4 |
|  |  | 648 | N | 0/4 |
|  |  | 586 | Y | 0/4 |
|  |  | 581 | N | 0/4, 0/4 |
|  |  | 547 | Y | 0/4, 0/4 |
|  |  | 700 | N | 0/4 |
|  |  | 700 | N | 0/4 |
|  |  | 700 | N | 0/4 |
| B351-3  662 DPI  4/12 RTQ score | Tg66 | 532 | Y | 1/4, 0/4 |
|  |  | 700 | N | 0/4 |
|  |  | 700 | N | 0/4 |
|  |  | 608 | N | 0/4 |
|  |  | 700 | N | 0/4 |
|  |  | 700 | N | 0/4 |
|  |  | 559 | N | 0/4 |
|  |  | 591 | N | 0/4 |
|  |  | 700 | N | 0/4 |
|  |  | 700 | N | 0/4 |
|  |  | 700 | N | 0/4 |
|  |  | 700 | N | 0/4 |
|  | Tg33 | 700 | N | 0/4 |
|  |  | 700 | N | 0/4 |
|  |  | 700 | N | 0/4 |
|  |  | 700 | N | 0/4 |
|  |  | 700 | N | 0/4 |
|  |  | 700 | Y | 0/4 |
|  |  | 700 | N | 0/4 |
|  |  | 700 | Y | 1/4 |
|  |  | 700 | N | 0/4 |
|  |  | 587 | N | 0/4 |
| B377-4  710 DPI  14/24 RTQ score | Tg66 | 694 | N | 0/4 |
|  |  | 700 | N | 0/4 |
|  |  | 700 | N | 0/4 |
|  |  | 700 | N | 0/4 |
|  |  | 452 | N | 0/4 |
|  |  | 694 | Y | 0/4 |
|  |  | 700 | N | 0/4 |
|  |  | 700 | N | 0/4 |
|  |  | 700 | N | 0/4 |
|  |  | 516 | N | 0/4 |
|  |  | 581 | N | 0/4 |
|  |  | 700 | N | 0/4 |
|  | Tg33 | 378 | N | 0/4 |
|  |  | 700 | N | 0/4 |
|  |  | 700 | N | 1/8 |
|  |  | 700 | N | 1/4 |
|  |  | 487 | N | 0/4 |
|  |  | 481 | N | 0/4 |
|  |  | 700 | N | 0/4 |
|  |  | 700 | N | 0/4 |
|  |  | 635 | N | 0/4 |
|  |  | 498 | N | 0/4, 0/4 |
|  |  | 635 | N | 1/4 |
| B378-4  717 days old  0/4 RTQ score | Tg66 | 622 | N | 1/4 |
|  |  | 629 | N | 0/4 |
|  |  | 629 | N | 0/4 |
|  |  | 673 | N | 0/4 |
|  |  | 673 | N | 0/4 |
|  |  | 469 | N | 0/4 |
|  |  | 628 | N | 0/4 |
|  |  | 628 | Y | 0/4 |
|  | Tg33 | 572 | N | 0/4 |
|  |  | 572 | N | 0/4 |
|  |  | 617 | N | 0/4, 0/4 |
|  |  | 700 | N | 0/4 |
|  |  | 700 | N | 0/4 |
|  |  | 677 | N | 0/4 |
|  |  | 677 | N | 3/4, 0/4, 0/4 |
| B348-3  635 days old  0/4 RTQ score | Tg66 | 700 | N | 0/4 |
|  |  | 700 | N | 0/4 |
|  |  | 657 | N | 0/4 |
|  |  | 671 | Y | 0/4 |
|  |  | 679 | Y | 0/4 |
|  |  | 700 | N | 0/4 |
|  |  | 700 | N | 0/4 |
|  |  | 700 | N | 1/4 |
|  | Tg33 | 679 | N | 0/4 |
|  |  | 679 | N | 0/4 |
|  |  | 700 | N | 0/4 |
|  |  | 700 | N | 0/4 |
|  |  | 700 | N | 0/4 |
|  |  | 700 | N | 1/4 |
|  |  | 700 | N | 1/4 |
| B354-4  635 days old  0/4 RTQ score | Tg66 | 700 | Y | 0/4 |
|  |  | 612 | N | 0/4 |
|  |  | 694 | Y | 0/4 |
|  |  | 668 | N | 0/4 |
|  |  | 668 | Y | 0/4 |
|  |  | 700 | Y | 0/4 |
|  |  | 700 | N | 0/4 |
|  |  | 700 | Y | 0/4 |
|  |  | 700 | Y | 0/4 |
|  |  | 700 | Y | 0/4 |
|  | Tg33 | 567 | N | 1/4, 1/4, 0/4 |
|  |  | 553 | N | 0/4 |
|  |  | 553 | N | 0/4 |
|  |  | 595 | N | 0/4 |
|  |  | 700 | Y | 0/4 |
|  |  | 700 | Y | 0/4 |
|  |  | 700 | Y | 0/4 |
|  |  | 700 | Y | 0/4 |
|  |  | 700 | Y | 0/4 |
|  |  | 700 | Y | 0/4 |
| B380-1  626 days old  0/4 RTQ score | Tg66 | 480 | N | 0/4 |
|  |  | 520 | Y | 0/4 |
|  |  | 700 | N | 0/4 |
|  |  | 700 | N | 0/4 |
|  |  | 700 | N | 0/4 |
|  |  | 700 | N | 0/4 |
|  |  | 700 | N | 0/4 |
|  |  | 700 | N | 0/4 |
|  |  | 700 | N | 0/4 |
|  | Tg33 | 700 | N | 0/4 |
|  |  | 700 | N | 0/4 |
|  |  | 700 | N | 0/4 |
|  |  | 700 | N | 0/4 |
|  |  | 700 | N | 1/4 |
|  |  | 603 | N | 1/4 |
|  |  | 603 | N | 0/4 |
|  |  | 519 | N | 1/4 |
| B351-1  662 days old  0/4 RTQ score | Tg66 | 463 | N | 0/4 |
|  |  | 497 | N | 0/4 |
|  |  | 586 | N | 0/4 |
|  |  | 586 | N | 0/4 |
|  |  | 505 | N | 0/4 |
|  |  | 653 | Y | 0/4 |
|  |  | 496 | N | 0/4 |
|  |  | 576 | N | 0/4 |
|  |  | 622 | N | 0/4 |
|  |  | 622 | N | 0/4 |
|  | Tg33 | 404 | N | 0/4 |
|  |  | 576 | N | 0/4 |
|  |  | 700 | N | 0/4 |
|  |  | 502 | N | 0/4 |
|  |  | 700 | N | 0/4 |
|  |  | 447 | Y | 1/4, 0/4 |
|  |  | 700 | N | 0/4 |
|  |  | 700 | N | 0/4 |
|  | | Days Old |  | |
| Uninoculated | Tg66 | 735 | N | 0/4, 0/4 |
|  |  | 735 | N | 0/4, 0/4 |
|  |  | 508 | N | 0/4, 0/4, 0/8, 0/4, 0/4, 0/8, 0/8, 0/4 |
|  |  | 690 | Y | 0/4 |
|  |  | 690 | N | 0/4 |
|  |  | 690 | N | 0/4 |
|  |  | 257 | N | 1/4 |
|  |  | 257 | N | 0/4 |
|  |  | 257 | N | 0/4 |
|  | Tg33 | 572 | N | 0/4, 0/4 |
|  |  | 572 | N | 0/8 |
|  |  | 288 | N | 0/4 |
|  |  | 481 | N | 1/4, 0/4, 0/4, 0/4 |
|  |  | 481 | N | 0/4, 0/4, 0/4, 0/4, 0/4, 0/4, 0/8 |

^1^ Mice that showed a decrease in body condition (weight loss), weakness and/or neurologic signs (ataxia, gait abnormalities or balance issues) were classified as positive for clinical signs.

^2^ The RT-QuIC assay was used to screen all second passage mice for prion seeding activity. The number of positive wells over the number of wells tested is shown. Mice were scored positive if ≥ 50% of the assay wells were positive. Some mice were tested on more than one plate, and these data are separated by commas. Positive controls were run on each assay plate and were consistently 100% positive (not shown in table).
