# Supplementary material for: Second passage experiments of chronic wasting disease in transgenic mice overexpressing human prion protein
Source: Vet Res. 2022 Dec 16;53:111. doi: 10.1186/s13567-022-01130-0 (PMC9758843; doi:10.1186/s13567-022-01130-0)
Supplement: Supplementary file 2 — Additional file 2. Detailed information for individual clearance study mice. Additional file 2 includes a table that provides RT-QuIC assay individual well data for transgenic mice included in the CWD clearance study. Data from three strains of transgenic mice inoculated with CWD are shown at time-points from 7 to 675 days post-inoculation [30]. [file 13567_2022_1130_MOESM2_ESM.docx]

**Additional file 2. Detailed information for individual clearance study mice.**

| **Inoculum** | **Strain** | **dpi** | **Mouse ID** | **RT-QuIC Score^1^** |
| --- | --- | --- | --- | --- |
| WTD-1 | Tg66 | 7 | C068-4 | 4/4 |
|  |  |  | C064-1 | 4/4 |
|  |  |  | C064-2 | 4/4 |
|  |  |  | C064-3 | 4/4 |
|  |  | 28 | C065-1 | 3/4 |
|  |  |  | C065-2 | 4/4 |
|  |  |  | C065-3 | 4/4 |
|  |  |  | C065-4 | 3/4 |
|  |  | 90 | C067-1 | 1/8 |
|  |  |  | C067-2 | 0/8 |
|  |  |  | C067-3 | 1/8 |
|  |  |  | C067-4 | 1/8 |
|  |  | 180 | C066-1 | 0/8 |
|  |  |  | C066-2 | 0/8 |
|  |  |  | C066-3 | 0/8 |
|  |  |  | C066-4 | 0/8 |
|  |  | 360 | C063-1 | 0/8 |
|  |  |  | C063-2 | 0/8 |
|  |  |  | C069-1 | 0/8 |
|  |  |  | C069-2 | 2/8 |
|  |  |  | C069-3 | 0/8 |
|  |  |  | C069-4 | 2/8 |
|  |  |  | C069-5 | 0/8 |
|  |  | 675 | C062-1 | 5/16 |
|  |  |  | C062-2 | 8/16 |
|  |  |  | C062-3 | 9/16 |
|  |  |  | C062-4 | 0/16 |

| Inoculum | Strain | dpi | Mouse ID | RT-QuIC Score |
| --- | --- | --- | --- | --- |
| WTD-1 | PrPKO | 7 | NL961 | 4/4 |
|  |  |  | NL962 | 4/4 |
|  |  |  | NL963 | 4/4 |
|  |  |  | NL964 | 4/4 |
|  |  | 28 | NL919 | 4/4 |
|  |  |  | NL920 | 7/8 |
|  |  |  | NL921 | 3/4 |
|  |  |  | NL922 | 3/4 |
|  |  | 90 | NL916 | 1/8 |
|  |  |  | NL917 | 1/8 |
|  |  |  | NL918 | 0/8 |
|  |  |  | NL926 | 0/8 |
|  |  | 180 | NL879 | 0/8 |
|  |  |  | NL881 | 0/8 |
|  |  |  | NL923 | 0/8 |
|  |  |  | NL924 | 0/8 |
|  |  | 360 | NL957 | 1/8 |
|  |  |  | NL960 | 0/8 |
|  |  |  | C076-1 | 0/8 |
|  |  |  | C076-2 | 0/8 |
|  |  | 675 | NL956 | 0/12 |
|  |  |  | NL958 | 0/12 |
|  |  |  | NL959 | 0/12 |
|  |  |  | NL929 | 0/12 |

| Inoculum | Strain | dpi | Mouse ID | RT-QuIC Score |
| --- | --- | --- | --- | --- |
| Elk-2 | Tg66 | 28 | C267-1 | 6/8 |
|  |  |  | C267-2 | 4/8 |
|  |  |  | C270-1 | 6/8 |
|  |  |  | C270-2 | 5/8 |
|  |  | 90 | C266-1 | 1/12 |
|  |  |  | C266-2 | 2/12 |
|  |  |  | C266-3 | 3/12 |
|  |  |  | C266-4 | 4/12 |
|  |  | 180 | C264-1 | 0/8 |
|  |  |  | C264-2 | 0/8 |
|  |  |  | C264-3 | 0/8 |
|  |  |  | C268-1 | 0/8 |
|  |  |  | C268-2 | 1/8 |
|  |  |  | C268-3 | 0/8 |
|  |  | 675 | C265-1 | 0/8 |
|  |  |  | C265-2 | 0/8 |
|  |  |  | C269-1 | 0/8 |
|  |  |  | C269-2 | 0/8 |
|  |  |  | C269-3 | 0/8 |

| Inoculum | Strain | dpi | Mouse ID | RT-QuIC Score |
| --- | --- | --- | --- | --- |
| Elk-2 | PrPKO | 7 | JP362 | 1/4 |
|  |  |  | JP387 | 4/4 |
|  |  |  | JP247 | 1/4 |
|  |  |  | JP248 | 2/4 |
|  |  | 28 | JP370 | 1/8 |
|  |  |  | JP371 | 2/8 |
|  |  |  | JP368 | 0/8 |
|  |  |  | JP369 | 0/8 |
|  |  | 90 | JP376 | 1/8 |
|  |  |  | JP378 | 0/8 |
|  |  |  | JP366 | 3/8 |
|  |  |  | JP367 | 2/8 |
|  |  | 180 | JP244 | 0/8 |
|  |  |  | JP245 | 0/8 |
|  |  |  | JP246 | 0/8 |
|  |  |  | JP374 | 1/8 |
|  |  | 360 | JP383 | 0/8 |
|  |  |  | JP384 | 0/8 |
|  |  |  | JP385 | 1/8 |
|  |  |  | JP358 | 0/8 |
|  |  |  | JP359 | 0/8 |
|  |  |  | JP360 | 0/8 |
|  |  |  | JP373 | 1/8 |
|  |  | 675 | JP363 | 1/8 |
|  |  |  | JP364 | 0/8 |
|  |  |  | JP365 | 0/8 |
|  |  |  | JP379 | 0/8 |
|  |  |  | JP380 | 0/8 |
|  |  |  | JP381 | 0/8 |
|  |  |  | JP382 | 2/8 |

| Inoculum | Strain | dpi | Mouse ID | RT-QuIC |
| --- | --- | --- | --- | --- |
| WTD-1 | Tg33 | 7 | NM900 | 4/4 |
|  |  |  | NM901 | 4/4 |
|  |  |  | NM902 | 4/4 |
|  |  |  | NM890 | 4/4 |
|  |  | 28 | NM896 | 4/4 |
|  |  |  | NM886 | 4/4 |
|  |  |  | NM887 | 4/4 |
|  |  |  | NM888 | 4/4 |
|  |  | 90 | NM865 | 8/8 |
|  |  |  | NM867 | 8/8 |
|  |  |  | NM857 | 8/8 |
|  |  |  | NM858 | 8/8 |
|  |  | 180 | NM848 | 4/4 |
|  |  |  | NM849 | 4/4 |
|  |  |  | NM868 | 4/4 |
|  |  |  | NM851 | 4/4 |
|  |  |  | NM852 | 4/4 |
|  |  |  | NM854 | 4/4 |
| Elk-2 |  | 7 | NM903 | 4/4 |
|  |  |  | NM904 | 4/4 |
|  |  |  | NM905 | 4/4 |
|  |  |  | NM906 | 4/4 |
|  |  | 28 | NM899 | 4/4 |
|  |  |  | NM893 | 4/4 |
|  |  |  | NM894 | 4/4 |
|  |  |  | NM895 | 4/4 |
|  |  | 90 | NM863 | 8/8 |
|  |  |  | NM864 | 8/8 |
|  |  |  | NM860 | 8/8 |
|  |  |  | NM861 | 8/8 |
|  |  | 180 | NM870 | 4/4 |
|  |  |  | NM880 | 4/4 |
|  |  |  | NM875 | 4/4 |
|  |  |  | NM876 | 4/4 |

| Inoculum | Strain | Days Old | Mouse ID | RT-QuIC Score |
| --- | --- | --- | --- | --- |
| None | PrPKO | 127 | NL849 | 2/12 |
|  |  | 127 | NL850 | 0/4 |
|  |  | 127 | NL851 | 0/16 |
|  |  | 127 | NL852 | 1/16 |
|  |  | 127 | NL853 | 0/8 |
|  | Tg66 | 257 | C244-1 | 1/4 |
|  |  | 257 | C244-2 | 0/4 |
|  |  | 257 | C244-3 | 0/4 |
|  |  | 735 | C242-2 | 0/8 |
|  |  | 735 | C242-3 | 0/8 |
|  |  | 508 | C243-1 | 0/44 |
|  |  | 690 | C243-2 | 0/4 |
|  |  | 690 | C243-3 | 0/4 |
|  |  | 690 | C243-4 | 0/4 |
|  | Tg33 | 481 | C247-1 | 0/4 |
|  |  | 481 | C247-2 | 0/24 |
|  |  | 572 | NM343 | 0/4 |

^1^ The RT-QuIC assay was used to screen all mice for prion seeding activity. The number of positive wells over the number of wells tested is shown. Mice were scored positive if ≥ 50% of the assay wells were positive. In addition to the tg33 inoculated mice shown above, additional positive controls were run on each assay plate and were consistently 100% positive (not shown in table).
